# Multi-Target Radar Speed Meter Failure Mechanism Research

**DOI:** 10.3390/s26103209

**Published:** 2026-05-19

**Authors:** Lei Luo, Xin Jiang, Jianwen Shao, Cheng Chen, Cunbin Zhao, Xin Zhang, Xiaomin Shen

**Affiliations:** 1 Zhejiang Institute of Quality Sciences, Hangzhou 310018, China; luoleinuaa@163.com (L.L.); 13777358267@126.com (X.J.); zhaocunbin@126.com (C.Z.); soominshim@163.com (X.S.); 2 Zhejiang Key Laboratory of Digital Precision Measurement Technology, Hangzhou 310018, China; 3 Zhejiang Key Laboratory of Acoustic Intelligent Sensing and Advanced Measurement, Hangzhou 310018, China; 4 School of Mechanical and Power Engineering, Nanjing Tech University, Nanjing 211816, China; chencheng88217@163.com

**Keywords:** multi-target radar speed measurement instruments, failure analysis, reliability, accelerated life test

## Abstract

Multi-target radar speed measuring instruments play a crucial role in monitoring speeding violations of multiple targets in multiple lanes, ensuring the safety, order, and efficient operation of traffic. In order to verify the stability of these instruments under long-term and complex working conditions, accelerated life testing was conducted, and the failure mechanisms of the radar were studied using optical and scanning electron microscopic analysis methods. The results indicated that at a test temperature of 85 °C and humidity of 79% RH, all five speed measuring specimens failed after testing for 55 days. The primary cause of failure was the malfunction of the microwave signal receiving chip in the radar speed module under the combination of temperature and humidity. The failure modes of the chip were mainly related to its structure, design, selection of soldering materials, and soldering process. This study provides references and insights for the reliability research of other metrological instrument products.

## 1. Introduction

Multi-target radar speed meters are expensive and have relatively high reliability levels. Traditional reliability life tests usually require large sample sizes, which are costly, or have long test cycles, which may delay the development process of multi-target radar speed meters. To quickly and effectively evaluate the reliability level of multi-target radar speed meters under the influence of various environmental stresses in actual working environments, accelerated life testing is needed. Currently, the main standards for reliability qualification testing in China are GJB-899A, “Reliability Qualification and Acceptance Test” [1], and GB 5080.7, “Verification Test Scheme for Failure Rate and Mean Time Between Failures under Constant Failure Rate Assumption” [2]. The reliability qualification test plans in GJB-899A are for products with an exponential life distribution. The provided timed censored test plans require relatively large sample sizes and long test times, while short-term timed censored test plans have relatively high producer and consumer risk. GB 5080.7 uses mean time between failures as the main indicator for reliability testing, recording accumulated test time under the condition of no failures. It requires multiple prototypes simultaneously, and the test cycle is usually long, resulting in high costs for enterprises. In practical engineering, considering the development cycle and cost, using GJB-899A and GB 5080.7 for reliability testing of multi-target radar speed meters is not the optimal solution.

Accelerated life testing (ALT), currently the most widely used product reliability acceleration test method [3,4], is used in reliability tests in various fields such as weaponry, mechanical products, and medicine [5,6]. The national standard GB 2689.1, “Constant Stress Life Test and Accelerated Life Test Method”, is widely used as a standard test method [7]. GB/T 17215.9311-2017, “Electrical Measuring Equipment Part 311: Accelerated Reliability Test for Temperature and Humidity”, is mainly suitable for electrical measuring equipment represented by electronic watt-hour meters, but it has the problem that some failure modes may not be detected [8].

Considering preliminary test results and actual conditions, the reliability of multi-target radar speed meters is relatively high. The test time required for failures to occur during testing is long, requiring reliability life assessment under small-sample or even zero-failure data conditions. To save test costs and shorten test time, the constant-stress fixed-number censored test method was chosen to conduct accelerated life tests on multi-target radar speed meters. Based on the analysis of historical failure data of similar single-target radar speed meters, using the Failure Modes, Effects, and Criticality Analysis (FMECA) method combining function and hardware, it was determined that the main factors affecting the reliability of multi-target radar speed meters are temperature and humidity, and the main faulty module is the microwave chip in the speed measurement module [9,10].

Optical microscopy (OM) and scanning electron microscopy (SEM) play important roles in the field of chip failure analysis. Optical microscopes are widely used for observing the macroscopic structure of chip surfaces, such as package outlines, pin arrangements, etc., due to their simple operation and low cost, enabling rapid discovery of obvious surface issues like scratches, oxidation, or corrosion [11]. Scanning electron microscopes are often used to observe the microstructure on the chip surface and shallow interior, such as metal wiring, brazed joints, etc., and can analyze the elemental composition of chip lines and pins, and solder through energy dispersive spectroscopy (EDS) functions to determine factors leading to failure, such as impurity mixing or elemental anomalies [12,13]. This study combines accelerated life testing and microscopic analysis methods to investigate the failure causes of multi-target radar speed meters and analyze and verify their failure mechanisms, aiming to provide reference and practical experience for reliability research on other microwave measuring instruments.

To provide a theoretical basis for the observed failure mechanisms, we refer to established physics-of-failure models. The combined effect of temperature and humidity on electronic component reliability is often described by Peck’s model [14,15], while the low-cycle fatigue failure of solder joints under thermal stress is well captured by the Coffin–Manson relationship [16,17]. The subsequent reliability life prediction and assessment of the measurement instruments under ALT, as well as the analysis of solder joint performance degradation, will be based on this theoretical framework.

Table 1 shows that radar speed meters from different manufacturers have similar operating frequencies and basic principles, with most employing Frequency Modulation Continuous Wave (FMCW) technology. However, minor differences exist in aspects such as weather filtering and RF chip packaging. This implies that the failure mechanisms observed on a specific design (e.g., ADF chip solder degradation) are generally applicable, and the proposed analytical methods and corrective actions have broad applicability.

## 2. Experimental Methods

According to the test results from our institution’s type evaluation laboratory for fixed-angle radar speed meters, conducted in accordance with JJF 1335-2012, “Type Evaluation Program for Fixed-Angle Radar Speed Meters” [18], under vibration testing, shock testing, and alternating damp heat testing conditions, the radar speed meters generally did not exhibit significant failures. In preliminary exploratory tests, we also conducted temperature cycling experiments. By measuring and analyzing the voltage ripple of key modules in each prototype, apart from black-screen shutdowns, the output voltage of the key modules remained largely unchanged throughout the tests. This demonstrates that temperature and humidity stress variations have a minimal impact on the key modules of the prototypes.

**Figure 1:**
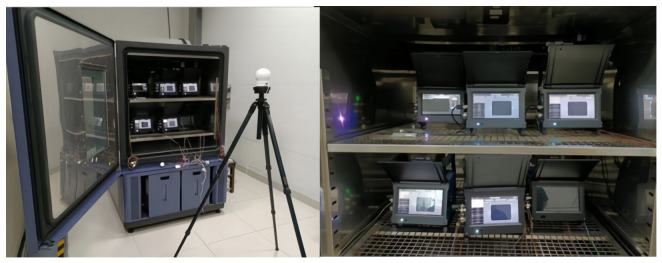
Accelerated life testing equipment and process.

Accelerated life tests were conducted on multi-target radar speed meters. Test equipment included the EW1070 temperature and humidity test chamber (measuring temperature range: −60 °C~150 °C, humidity: 20%~95%) from Guangzhou Wusuo Environmental Instruments Co., Ltd., Guangzhou, China, the key voltage acquisition unit (data acquisition card, acquisition rate 200 kS/s, 16-bit resolution), the temperature acquisition unit for critical component heat points (temperature and humidity sensor connection: first, calibrate the sensors monitoring critical heat-generating components, prototype temperature and humidity, and chamber temperature and humidity separately; the sensor supply voltage 24 V DC was supplied by the same regulated power supply), the host computer LabVIEW2021 data acquisition system, and the power supply. The field diagram of the reliability test platform is shown in Figure 1. Five MiDS-900 multi-target radar speed meters from Hangzhou Hikvision Digital Technology Co., Ltd., Hangzhou, China, were subjected to accelerated life testing at a test temperature of 85 °C and relative humidity of 79%. Failure was considered reached when the simulated speed measurement error exceeded the tolerance under these test conditions, including exceeding the required range of −4 km/h to 0 km/h, showing no response to speed measurement, fluctuating speed measurement readings, etc.

It should be noted that the speed measurement error determination in this study is based on the output results of the built-in multi-target speed measurement algorithm of the speedometer. This algorithm employs Fast Fourier Transform (FFT) combined with Constant False Alarm Rate (CFAR) detection to achieve multi-lane target velocity extraction. During testing, in addition to hardware failures, the algorithm may experience target miss detection, velocity ambiguity, and increased false alarm rates under extreme temperature and humidity conditions, leading to abnormal measurement results (e.g., value fluctuation, no response). Furthermore, simulated speed measurement is conducted using a standard speed simulator generating microwave signals of known frequency. In the test result determination, “speed measurement error” refers to the difference between the instrument reading and the standard value from the simulator, while “no response” indicates that the algorithm failed to effectively extract the target velocity. The above algorithm implementation details and measurement result determination criteria have been explicitly defined in the test protocol to ensure accurate data interpretation.

Microstructural analysis and chemical composition testing methods were used to analyze and test the internal structure, pins, and brazed joints of the chips before and after the test, as follows: A micro-cutter was used to separate the chip from the circuit board without damaging the chip’s pins and brazed joints. Surface grinding was used to remove the packaging material, followed by polishing to observe and analyze the structure and composition beneath the packaging layer. A micro-cutter was used to cut the chip in half, and cross-sectional analysis samples were prepared through mounting, grinding, and polishing. A Leica DM2700M optical metallurgical microscope (Wetzlar, Germany) was used to observe the surface and cross-sectional macromorphology of the chip. A JEOL JSM-6380LA scanning electron microscope (SEM) (Tokyo, Japan) and its accompanying JED-2300 energy-dispersive spectrometer (EDS) (Tokyo, Japan) were used to analyze its microstructure and chemical composition.

## 3. Results and Discussion

The data acquisition system (including voltage and temperature sensors) was primarily employed to monitor and verify the stability of the test conditions. Continuous monitoring confirmed that the supply voltage for the radar units remained within ±1% of the nominal 24V DC, and the temperature of key heating components stabilized within ±2 °C of the setpoint after an initial warm-up period. The stable monitored data confirm that the observed failures were indeed due to the long-term synergistic effect of temperature and humidity, rather than fluctuations in the test environment or power supply.

**Figure 2:**
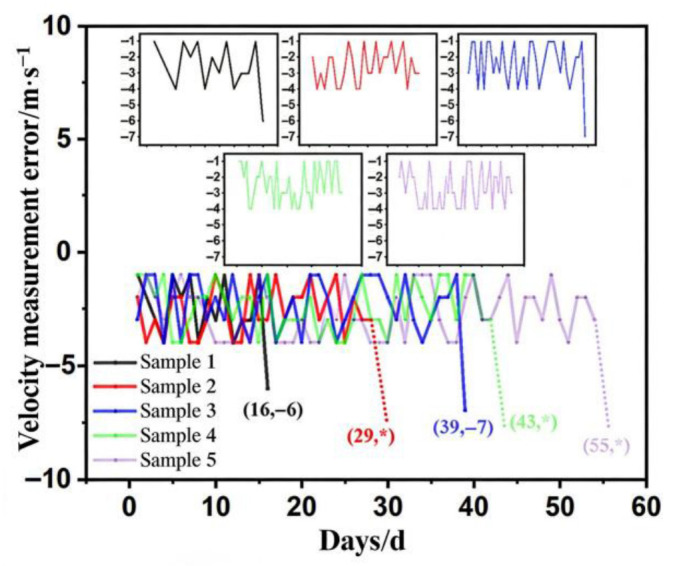
Error variation curve of specimens in accelerated life testing.

Under the test conditions (temperature T = 85 °C, humidity H = 79% RH), it took a total of 55 days for the number of failures among the five samples to reach five. The fault and failure data are shown in Table 2, and the speed measurement errors are shown in Figure 2, where * in samples 2 and 5 indicates sample failure due to no response from the speed measurement module, and * in sample 4 indicates sample failure due to fluctuating speed measurement readings. Around 10 days into the test, the simulated speed measurement errors for instruments 1 and 2 were −4 km/h and −3 km/h, respectively. Instrument 1 also experienced slow speed measurement response. Instruments 3, 4, and 5 all showed fading around the screen edges.

Around 20 days into the test, instruments 1 and 2 showed a leakage spot on the screen, and instrument 1 was judged as failed on day 16 due to speed measurement error out of tolerance (−6 km/h). Instruments 3, 4, and 5 showed fading screens. Instruments 2 and 3 also experienced slow speed measurement response, but their speed measurement errors were still within the tolerance range (−4 km/h and −2 km/h). Around 30 days into the test, instrument 3 experienced freezing and then a black screen, but speed measurement was still possible by restarting and using a wireless connection, with a speed measurement error of −4 km/h, still within tolerance. Instrument 4 showed a leakage spot on the screen and slow speed measurement response, but the speed measurement error was still within tolerance (−4 km/h). Instrument 5 showed unclear display on the screen, with a speed measurement error of −3 km/h at that time, within tolerance. On day 29 of the test, the leakage spot on instrument 2 expanded, and the simulated speed measurement had no response, leading to failure. The remaining three instruments continued the test. Instrument 3 was judged as failed on day 39 of the test due to speed measurement error (−7 km/h) out of tolerance. As the test progressed, the leakage spot on instrument 4 expanded, accompanied by slow speed measurement response. On day 43 of the test, two leakage spots appeared on the screen, and the simulated speed measurement readings fluctuated, preventing accurate speed measurement, leading to failure. During subsequent testing, instrument 5 experienced a black screen issue, but error speed measurement was still possible via wireless connection. At this time, the speed measurement error was −4 km/h, within tolerance. On day 51 of the test, the instrument experienced slow wireless connection response. By day 55, the wireless-connection simulated speed measurement had no response, leading to failure.

According to the results of Table 1, a two-parameter Weibull distribution, which is widely adopted in reliability engineering for describing the lifetime behavior of electronic products with wear-out failure mechanisms, was fitted to these data.

**Figure 3:**
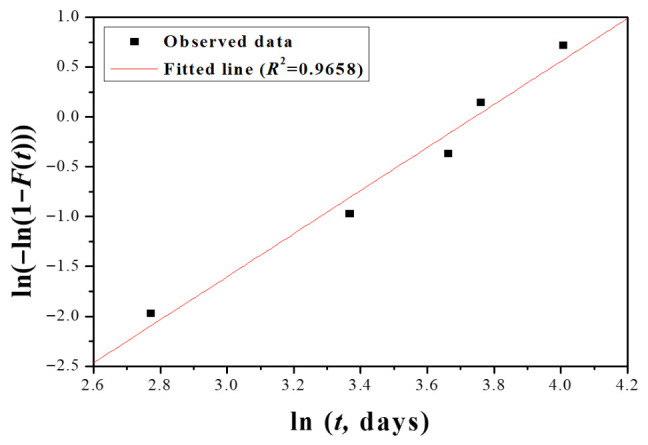
Weibull probability plot and fitting results.

The Weibull reliability function is defined as:

(1) $$R(t)=exp[-{\left(\frac{t}{\eta }\right)}^{\beta }]$$

where *β* is the shape parameter reflecting the failure mode, and *η* is the characteristic life. Parameter estimates and 95% confidence intervals (CIs) were obtained using the maximum likelihood estimation (MLE) combined with the bootstrap resampling method (1000 replications), which provides robust interval estimates for small sample sizes. The Weibull probability plot is provided in Figure 3, yielding a high correlation coefficient (*R*^2^ = 0.9658), confirming a good fit between the data and the Weibull distribution. The results are summarized in Table 3. The shape parameter *β* = 3.14 (95% CI: [2.10, 10.00]) indicates a wear-out failure mechanism, which is consistent with the observed gradual degradation phenomena (e.g., liquid leakage, screen dimming) in the accelerated test. The characteristic life *η* = 40.79 days (95% CI: [27.23, 50.76] days) represents the time at which the reliability drops to 36.8%. The mean time to failure (MTBF) under the test conditions was estimated to be 36.51 days.

The mean time to failure (MTBF) under the accelerated condition was calculated as 36.51 days.

**Figure 4:**
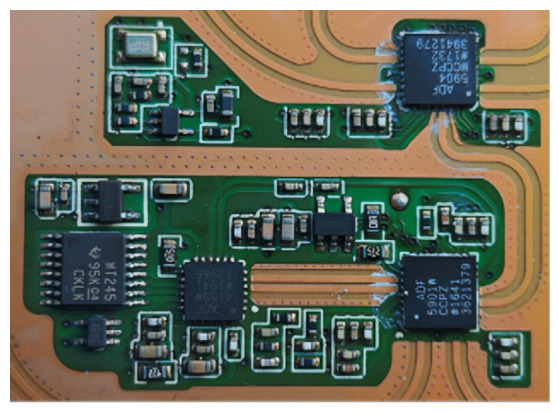
Circuit board of the multi-target radar speed measurement module.

After the accelerated life test, the radar speed measurement module circuit board was removed from the speed meter. As shown in Figure 4, the microwave chips on this circuit board mainly include an ADF5904WCCPZ four-channel radar microwave signal receiving chip, an ADF5901WCCPZ two-channel microwave signal transceiver chip, an ADF4159W microwave modulation and signal feedback chip, and a WT245 digital modem chip for microwave signal conversion and transmission.

**Figure 5:**
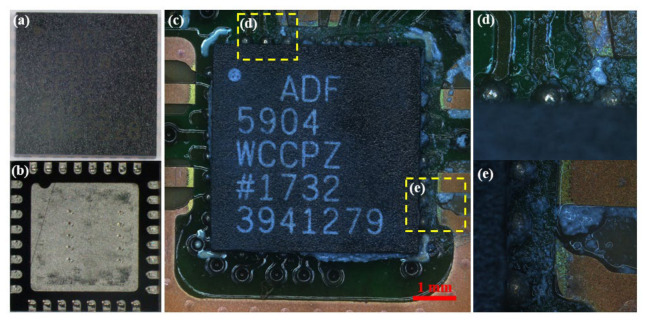
Surface morphology of ADF5904 chip before and after accelerated life test. (**a**) Chip package casing before test; (**b**) chip pads before test; (**c**) chip and circuit board after test; (**d**) upper brazed solder joint after test; (**e**) right brazed solder joint after test.

Figure 5 shows the surface macrographs of the ADF5904 chip before and after the test. Figure 5a shows the top view of the chip, mainly the package casing. Figure 5b shows the bottom view of the chip, revealing that this is a 32-pin chip, with 32 peripheral pads and a central exposed pad on the bottom. After the accelerated life test, no significant damage or cracking was observed on the chip package backplane. The metal luster of the pin connection points was darker, the copper color near the wire bond end of the copper wire was lighter, and the circuit board at this location showed debonding, but the traces showed no abnormality or exposure, as shown in Figure 5c–e.

**Figure 6:**
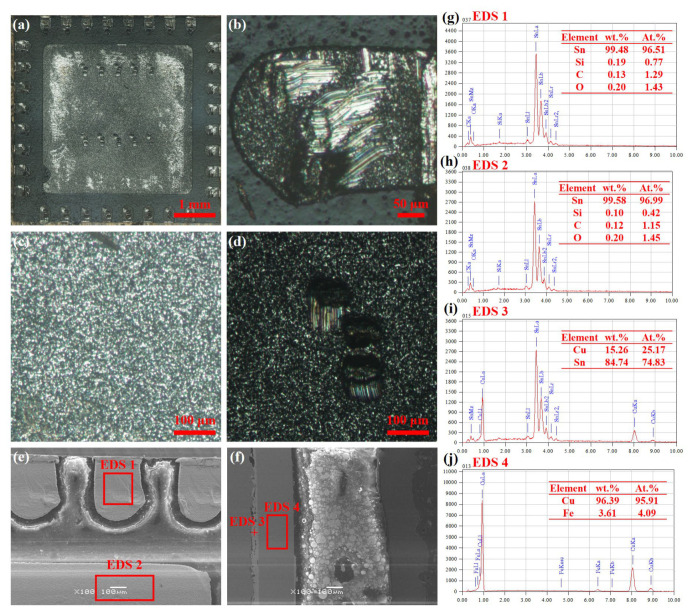
Microstructure and chemical composition of solder pads on ADF5904 chip before accelerated life test. (**a**) Pad macromorphology; (**b**) optical micrograph of pin solder;(**c**) optical micrograph of thermal pad; (**d**) optical micrograph of thermal pad wire encapsulation; (**e**) SEM image of pad; (**f**) cross-sectional SEM image of pad; (**g**) chemical composition of pin pad; (**h**) chemical composition of thermal pad; (**i**) chemical composition of thermal pad cross-section; (**j**) chemical composition of heat sink.

Figure 6 shows the high-magnification morphology and chemical composition of the ADF5904 chip pads before the test. Among them, the 32 peripheral pads were completely encapsulated, with no obvious missing solder or cold solder joints observed. The wire bundles were fully encapsulated, with no exposure found, as shown in Figure 6a,b. The central pad solder was uniform and flat, and none of the ten connection points to the internal chip showed obvious exposure, with complete wire sealing, as shown in Figure 6c,d. Figure 6e shows the SEM image of the chip. From the EDS results of the marked areas in Figure 6g,h, it can be seen that the pads were mainly sealed by brazing with Sn solder (lead-free), and no significant oxidation occurred. Figure 6f shows the cross-sectional micromorphology of the chip. Combined with the chemical composition of the marked areas in Figure 6i,j, it can be seen that the main content of the chip heat sink plate is Cu, and the bottom is a Sn-based thermal pad, used for connection with the circuit board thermal pad.

**Figure 7:**
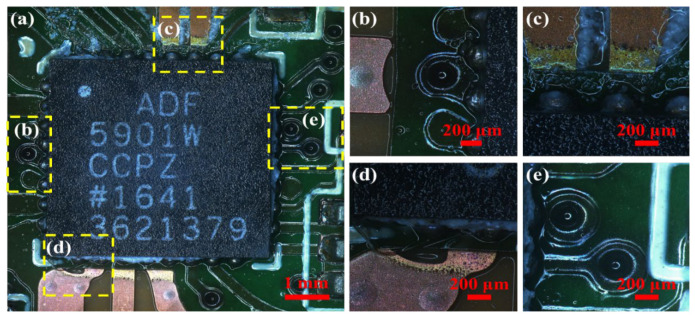
Morphology of solder pads on ADF5901 chip after accelerated life test. (**a**) Chip and circuit board; (**b**) left brazed solder joint of chip; (**c**) upper brazed solder joint of chip; (**d**) lower brazed solder joint of chip; (**e**) right brazed solder joint of chip after test.

Figure 7 shows the post-test surface macrographs of the ADF5901 chip. Its condition is similar to that of the ADF5904 chip, mainly characterized by loss of metallic luster at the pin solder joints after the temperature and humidity test, lighter copper color at the connection between the copper wire and the chip, and visible debonding of the circuit board at that location, but no abnormality or exposure of the traces.

**Figure 8:**
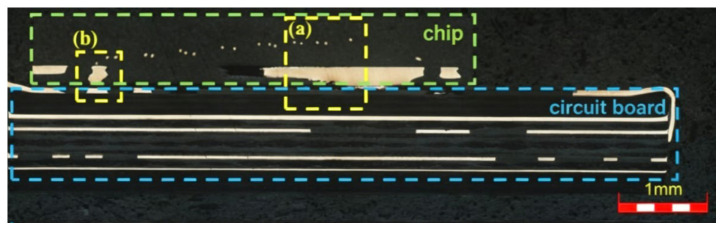
Cross-sectional morphology of ADF5904 chip circuit board after accelerated life test.

**Figure 9:**
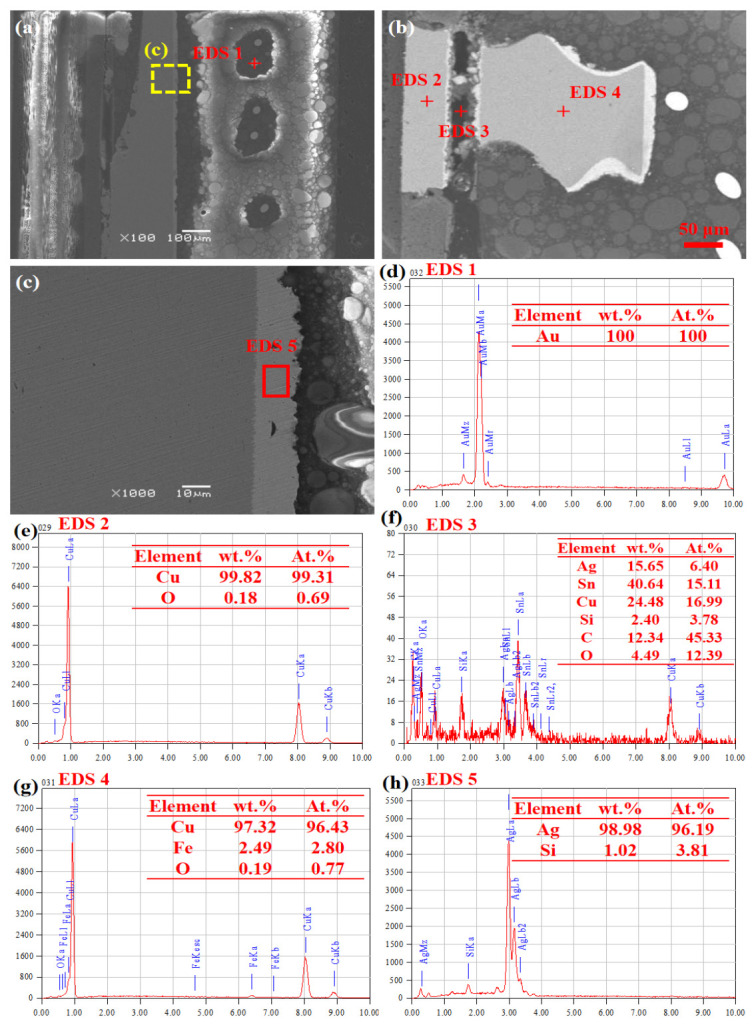
Cross-sectional microstructure and chemical composition of ADF5904 chip after accelerated life test. (**a**) Morphology of connecting wire and heat sink; (**b**) connection morphology between thermal pad and circuit board; (**c**) morphology of heat sink coating; (**d**) chemical composition of connecting wire; (**e**) chemical composition of circuit board pad; (**f**) chemical composition of solder layer; (**g**) chemical composition of heat sink; (**h**) chemical composition of heat sink coating.

**Figure 10:**
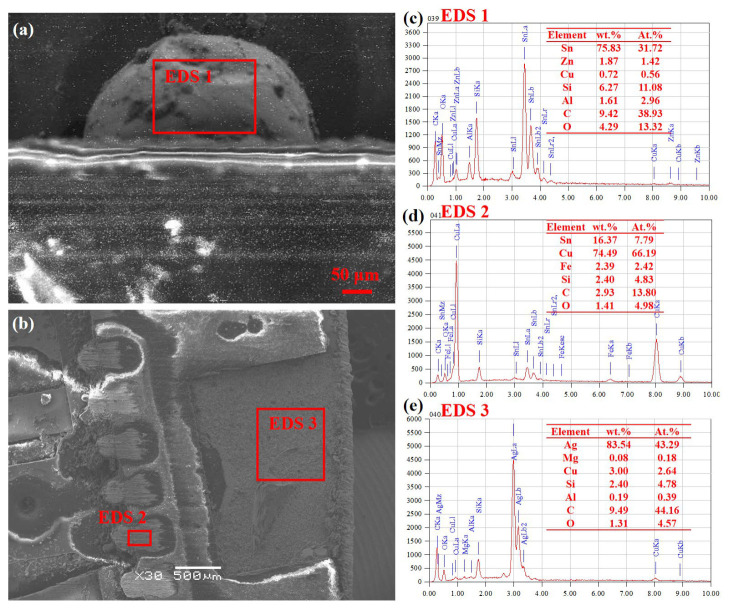
Microstructure and chemical composition of pin connectors and packaging shell of ADF5904 chip after accelerated life test. (**a**) Morphology of pin joint; (**b**) internal morphology of package shell; (**c**) chemical composition of pin joint; (**d**) internal chemical composition of pin pad; (**e**) internal chemical composition of package shell.

Figure 8 shows the optical micrograph of the cross-section of the ADF5904 chip circuit board. It can be observed that the solder joint between the thermal pad and the circuit board is discontinuous, with localized cold solder joints. To further observe the connection between the chip and the circuit board, the marked areas in Figure 8 were observed at high magnification for microstructure and chemical composition determination using SEM and EDS, as shown in Figure 9. In the right area of Figure 8, the chip thermal pad and PCB board are completely connected, with no obvious defects found in the solder joint, as shown in Figure 9a. Above the solder joint, there are distributed wire bundles with a circular cross-section whose main component is Au, as shown in Figure 9d. Above the Cu-based heat sink, a thermal coating layer of about 10 μm is visible, whose main component is Ag, as shown in Figure 9c,h. Figure 9b is the micrograph of the left area of Figure 8, showing discontinuity and observable voids in the solder joint between the chip thermal pad and the PCB board pad, indicating possible cracking at the connection interface, leading to a cold solder joint, which in turn causes localized poor heat dissipation or overheating of the chip. From Figure 9e–g, it can be seen that the PCB board pad is pure Cu, while the chip thermal pad contains a small amount of Fe. The solder is lead-free Sn-based solder, with a certain amount of Ag added to improve temperature adaptability. Combined with the accelerated life test conditions, thermal fatigue damage is speculated to be an important contributing factor to the failure of this chip. That is, under cyclic thermal expansion and contraction induced by temperature variation, the solder joint between the thermal pad and the PCB pad is subjected to repeated tensile and compressive stresses. During this process, microcracks may gradually initiate and propagate at the interfaces between the solder and the bonding pads, as well as at the phase boundaries of hard and brittle intermetallic compounds within the solder. However, it should be noted that a direct causal link between the observed solder degradation phenomena (Figure 9 and Figure 10) and the functional failure of the device has not been fully established. The solder oxidation, interfacial crack initiation, and degradation of the chip’s microwave-signal-receiving performance may represent parallel degradation processes or be indirectly associated.

Figure 10 shows the microstructure and chemical composition of the chip’s peripheral pin brazed joints and the interior of the center packaging plastic. From Figure 10a, it can be seen that the surface of the pin solder joint shows dark gray oxidation pits. Composition analysis (Figure 10c) reveals that the solder is mainly lead-free Sn-based solder, without the addition of Ag for better high-temperature adaptability. The oxygen content on the joint surface is relatively high, indicating that during the accelerated life test, the combined effect of high temperature and humidity promoted oxidation corrosion on the brazed joint surface, subsequently forming poorly conductive oxide or hydroxide films, thereby affecting its conductivity or stability. The normal cross-sectional morphology of the pin and solder joint is shown in Figure 10b. The solder is tightly connected to the pin pad, with no obvious cracks or dewetting found at the interface. Judging by the chemical composition (Figure 10d), no significant oxidation occurred inside the pin. Figure 10e shows the Ag-based coating above the Cu heat sink under the chip. Above it is resin-based adhesive material, hence containing small amounts of Si, C, and O.

**Figure 11:**
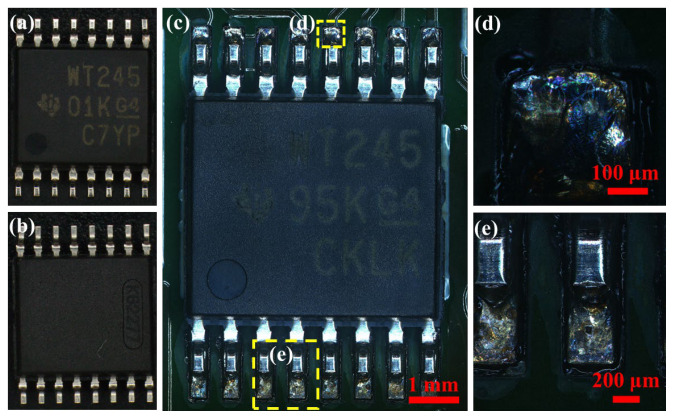
Surface morphology of WT245 chip before and after accelerated life test. (**a**) Chip package casing before test; (**b**) bottom view of chip before test; (**c**) chip and circuit board after test; (**d**) upper pin brazing after test; (**e**) lower pin brazing after test.

Figure 11 shows the macromorphology of the WT245 chip before and after the accelerated life test. The WT245 is 16-pin chip using conventional SOP packaging. Pins are led out from the corresponding two sides, and there is no thermal pad on the bottom, as shown in Figure 11a,b. After the test, no significant changes were observed macroscopically on this chip, but its pin joints showed a colorful luster, as shown in Figure 11c–e.

**Figure 12:**
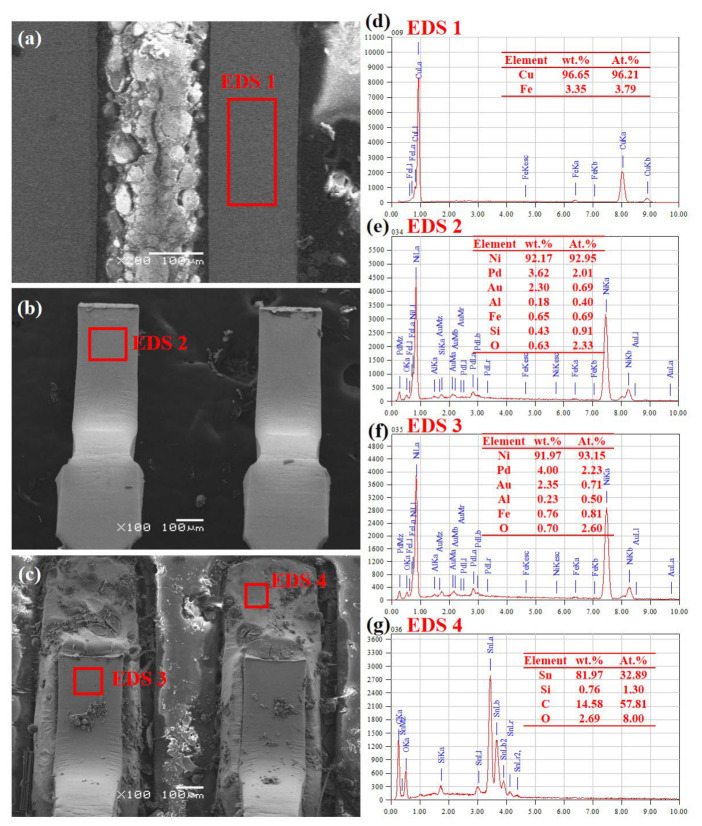
Microstructure and chemical composition of WT245 chip before and after accelerated life test. (**a**) Cross-sectional morphology of chip before test; (**b**) pin morphology of chip before test; (**c**) pin morphology of chip after test; (**d**) composition of chip heat sink before test; (**e**) pin composition of chip before test; (**f**) pin composition of chip after test; (**g**) pin solder layer composition of chip after test.

Figure 12a shows the cross-sectional microstructure of the chip before the test. Its heat sink is Cu, also containing a small amount of Fe (Figure 12d). The pins and chemical composition before the test are shown in Figure 12b,e. The outer layer is an electroplated Ni layer (internal pin is generally pure Cu according to the literature), containing small amounts of Au and Pd, which can improve thermal stability and chemical stability. This plating layer has good temperature and environmental adaptability. Its composition did not change significantly after the long-term test, as shown in Figure 12c,f. Figure 12g shows chemical composition after the test, whereby it can be seen that the solder is mainly lead-free Sn-based solder, with relatively high C and O content, possibly undergoing oxidation during the test.

## 4. Conclusions

(1) Under the accelerated life test conditions (test temperature T = 85 °C, humidity H = 79% RH), all five devices failed after 55 days. Failure types included speed measurement error exceeding 6 km/h, screen leakage spot expansion, and fluctuating or non-responsive simulated speed measurement readings.

(2) ADF-series chips generate significant heat and use LFCSP packaging to accelerate heat dissipation. The bottom thermal pad has a large area, resulting in a large soldering contact area. During the test, under the combined effect of temperature and humidity, the interface between the solder, thermal pad, and PCB pad is prone to cracking, leading to a gradual decrease in heat dissipation performance. This causes the chip to overheat, slowing down signal transmission and processing, resulting in thermal failure. Additionally, the peripheral pads of the chip are not protected by temperature- and humidity-resistant coatings. Under the combined effect of temperature and humidity, the solder joint surface undergoes oxidation corrosion, affecting its conductive function. If oxidation corrosion further intensifies, external oxides (O_2_, H_2_O, etc.) may diffuse into the pad interior, forming oxides or ionic compounds, leading to abnormal conductivity and significantly affecting the chip’s transmission and reception functions.

(3) WT-series chips use SOP packaging. Their pins are protected by an electroplated Ni layer, with elements such as Au and Pd added to improve high-temperature resistance and oxidation resistance. Although the solder gradually oxidizes during the test, under the protection of the coating, the pins can maintain a stable connection with the PCB board for a long time, reducing the risk of environmental impact.

In summary, the failure of the multi-target radar speed meters is mainly due to the failure of the ADF-series chips in the radar speed measurement module under the combined influence of temperature and humidity. The failure modes of the chips are primarily related to the chip’s own structure and design, selection of soldering materials, and brazing process.

Based on the above failure analysis results, the following corrective actions and recommendations are proposed: (1) Optimize the chip packaging design by replacing the current Sn-based solder with Sn–Ag–Cu lead-free solder featuring better high-temperature and high-humidity resistance, and add underfill to alleviate thermal stress concentration. (2) Perform structural optimization at the heat-sink-to-PCB connection interface, considering the use of flexible substrates or thermal-expansion-coefficient-matched materials. (3) Add conformal coating during circuit board assembly processes to improve moisture and oxidation resistance.

## Figures and Tables

**Table 1:** Comparison of multi-target radar speed meter technologies from different manufacturers.

Manufacturer	Model	Technology	Frequency (GHz)	Weather Filtering	Key Packaging for RF Chip
Manufacturer A	MiDS-900	FMCW	24.15	Digital (FFT-based)	LFCSP (ADF series)
Manufacturer B	Model X-1	CW	24.125	Analog filtering	QFN
Manufacturer C	Model R-2	FMCW	24.15	Digital (FFT-based)	SOP (WT series-like)
Manufacturer D	Model Z-2	FMCW	24.15	Digital (FFT-based)	LFCSP (ADF series)

**Table 2:** Fault and failure data (T = 85 °C, H = 79% RH, n = 5).

Sample No.	Test Time (Days)	Failure Phenomenon
1	8	Slow speed measurement response; speed measurement error −4 km/h
	13	One LCD leakage spot appeared; speed measurement error −4 km/h
	16	Speed measurement error −6 km/h; failure
2	9	Speed measurement error −3 km/h
	13	Slow speed measurement response; speed measurement error −4 km/h
	18	One LCD leakage spot appeared; speed measurement error −3 km/h
	29	Leakage spot expanded; no response in simulated speed measurement; failure
3	10	Screen edges became lighter
	15	Slow response in speed measurement mode; speed measurement error −2 km/h
	16	Overall screen became lighter with blurred display; speed measurement error −1 km/h
	20	System crash; manually restarted and recovered; speed measurement error −4 km/h
	24	Black screen; simulated speed measurement via wireless connection showed error −4 km/h
	39	Speed measurement error −7 km/h; failure
4	9	Screen edges became lighter
	18	Progressive lightening around screen edges; speed measurement error −3 km/h
	26	One LCD leakage spot appeared; slow speed measurement response; speed measurement error 4 km/h
	35	Leakage spot expanded; slow speed measurement response; speed measurement error −3 km/h
	43	Two LCD leakage spots appeared; unstable simulated speed indication; failure
5	10	Screen edges became lighter
	17	Overall screen gradually became lighter; speed measurement error −4 km/h
	26	Blurred display; speed measurement error −3 km/h
	38	Black screen; simulated speed measurement via wireless connection showed error −4 km/h
	51	Slow wireless connection response; speed measurement error −4 km/h
	55	No response in simulated speed measurement via wireless connection; failure

**Table 3:** Weibull distribution parameter estimates with 95% confidence intervals.

Parameter	Estimate	95% Confidence Interval
*β*	3.14	[2.10, 10.00]
*η* (days)	40.79	[27.23, 50.76]

## Data Availability

The data used to support the findings of this study are included within the article.

## References

[1] (2009). Reliability Qualification and Acceptance Testing.

[2] (1986). Equipment Reliability Testing—Verification Test Plans for Failure Rate and Mean Time Between Failures Under the Assumption of Constant Failure Rate.

[3] Zhu Y., Elsayed E.A. (2013). Design of accelerated life testing plans under multiple stresses. Nav. Res. Logist..

[4] Wang L.L., Chen Y. (1994). Statistical analysis of life tests and accelerated tests under log-normal distribution timed censored samples. Acta Math. Appl. Sin..

[5] Zhao J.J., Zou J.X., Song X., Huang J.X. (2002). Accelerated life test study on residual life calculation model of friction welded joints for boiler serpentine tubes. Proc. CSEE.

[6] Xu X.L., Fei H.L. (1999). Statistical analysis of step-stress accelerated life tests under Weibull distribution. Oper. Res. Trans..

[7] Mao S.S., Wang L.L. (1997). Accelerated Life Testing.

[8] Yu S.D., Zheng Z.X., Zhang Q.M. (2022). Analysis and discussion on accelerated life test standards for electricity meters. Electron. Prod. Reliab. Environ. Test..

[9] Song L. (2019). Research on environmental adaptability protection methods for radar equipment in coastal areas. Mod. Inf. Technol..

[10] Zhang M., Li J., He Q., Wen P., Wang B.H. (2024). Research on failure modes and quality assurance of microwave multi-chip modules. Electron. Prod. Reliab. Environ. Test..

[11] Ming Z., Ling X., Bai X., Zong B. (2016). A review of the technology and process on integrated circuits failure analysis applied in communications products. J. Phys. Conf..

[12] Liu Y., Sun F., Zhang H., Xin T., Yuan C.A., Zhang G. (2015). Interfacial reaction and failure mode analysis of the solder joints for flip-chip LED on ENIG and Cu-OSP surface finishes. Microelectron. Reliab..

[13] Gopinath S.C.B., Ramanathan S., Yasin M.N.M., Razak M.I.S., Ismail Z.H., Salleh S., Sauli Z., Malarvili M., Subramaniam S. (2022). Failure analysis on silicon semiconductor device materials: Optical and high-resolution microscopic assessments. J. Mater. Res. Technol..

[14] Peck D.S., Zierdt C. (1974). The reliability of semiconductor devices in the bell system. Proc. IEEE.

[15] Lu C.H., Zhang Y.J., Lu S.S., Sun Z.R., Zhang R.F., Liu L.Z. (2022). Performance and Life of Glass Fiber Reinforced PC Based on Hallberg-Peck Model. Plast. Ind..

[16] Coffin L.F. (1954). A study of the effects of cyclic thermal stresses on a ductile metal. Trans. ASME.

[17] Tauscher M., Lämmle S., Roos D., Wilde J. (2024). Bayesian calibration of ball grid array lifetime models for solder fatigue. Microelectron. Reliab..

[18] (2012). Program of Pattern Evaluation of Fixed-angle Radar Speed Measurement Devices.

